# 7-Ketocholesterol in disease and aging

**DOI:** 10.1016/j.redox.2019.101380

**Published:** 2019-11-14

**Authors:** Amelia Anderson, Angielyn Campo, Elena Fulton, Anne Corwin, W. Gray Jerome, Matthew S. O'Connor

## Abstract

7-Ketocholesterol (7KC) is a toxic oxysterol that is associated with many diseases and disabilities of aging, as well as several orphan diseases. 7KC is the most common product of a reaction between cholesterol and oxygen radicals and is the most concentrated oxysterol found in the blood and arterial plaques of coronary artery disease patients as well as various other disease tissues and cell types. Unlike cholesterol, 7KC consistently shows cytotoxicity to cells and its physiological function in humans or other complex organisms is unknown. Oxysterols, particularly 7KC, have also been shown to diffuse through membranes where they affect receptor and enzymatic function. Here, we will explore the known and proposed mechanisms of pathologies that are associated with 7KC, as well speculate about the future of 7KC as a diagnostic and therapeutic target in medicine.

## Highlights

• 7KC is mainly a nonenzymatically produced oxysterol known at the moment for its cytotoxicity.
• 7KC is implicated in many age-related diseases.
• 7KC appears to be a good target for disease, age related and otherwise.
• Removal of 7KC from the body could stall and/or reverse disease progression.

## Graphical abstract

**Figure:**
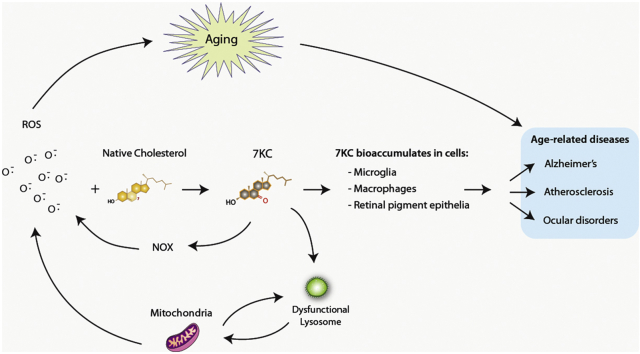

## 1 Introduction

Cholesterol, one of the most abundant and essential molecules in the body, creates functional membranes by influencing fluidity and allows cells to biosynthesize a variety of other important molecules. Cholesterols exist both inside and outside of the cell, as they are important components of all cellular membranes, but these and other nonpolar substances are transported in the plasma via lipoprotein particles (classified by hydrated density) which are otherwise insoluble in blood [1]. Low density lipoprotein (LDL) is the principal carrier of cholesterol to peripheral tissue. LDL is composed of a cholesterol, protein, and phospholipid shell with a core of cholesteryl esters and triglycerides. All of the components of LDL are susceptible to oxidation to produce an oxidized form of LDL (OxLDL). OxLDL has been linked to a variety of pathologies [[1], [2], [3], [4], [5], [6], [7], [8], [9]]. Oxidation of the cholesterol in LDL produces several oxidation products including 7KC, which is the most abundant oxysterol present in OxLDL [9,10]. We believe that it is important to distinguish between the effects of OxLDL and that of unsequestered 7KC, as many studies fail to account for this important difference in how 7KC interacts with the cell.

OxLDL is not the only source of 7KC within the body. 7KC can be produced endogenously by a series of oxidation steps or, much less commonly, enzymatic reactions [[11], [12], [13]]. It can also be ingested directly in food, however the liver is well equipped to process and rid the body of exogenous toxins, so 7KC is not acutely poisonous to ingest [14]. Endogenously produced, unsequestered 7KC can, on the other hand, wreak havoc inside of most cells. Unesterified 7KC can be found within membranes of organelles where it disrupts fluidity and signaling pathways, causing cellular damage via multiple stress-response pathways [[15], [16], [17], [18]]. These stress-response pathways induce a vicious cycle by increasing the population of reactive oxidative species (ROS), which in turn increases the oxidation of cholesterol and production of 7KC. Particularly in people with already-compromised cholesterol pathways, 7KC buildup can be overwhelming and cause significant damage to membranes, pathways, and overall cell function. In this review, we will discuss the chemistry and cell biological effects of 7KC and show how these attributes are critical contributors to a number of important diseases and to the aging process itself.

## 2 Chemistry of 7-Ketocholesterol

**Fig. 1:**
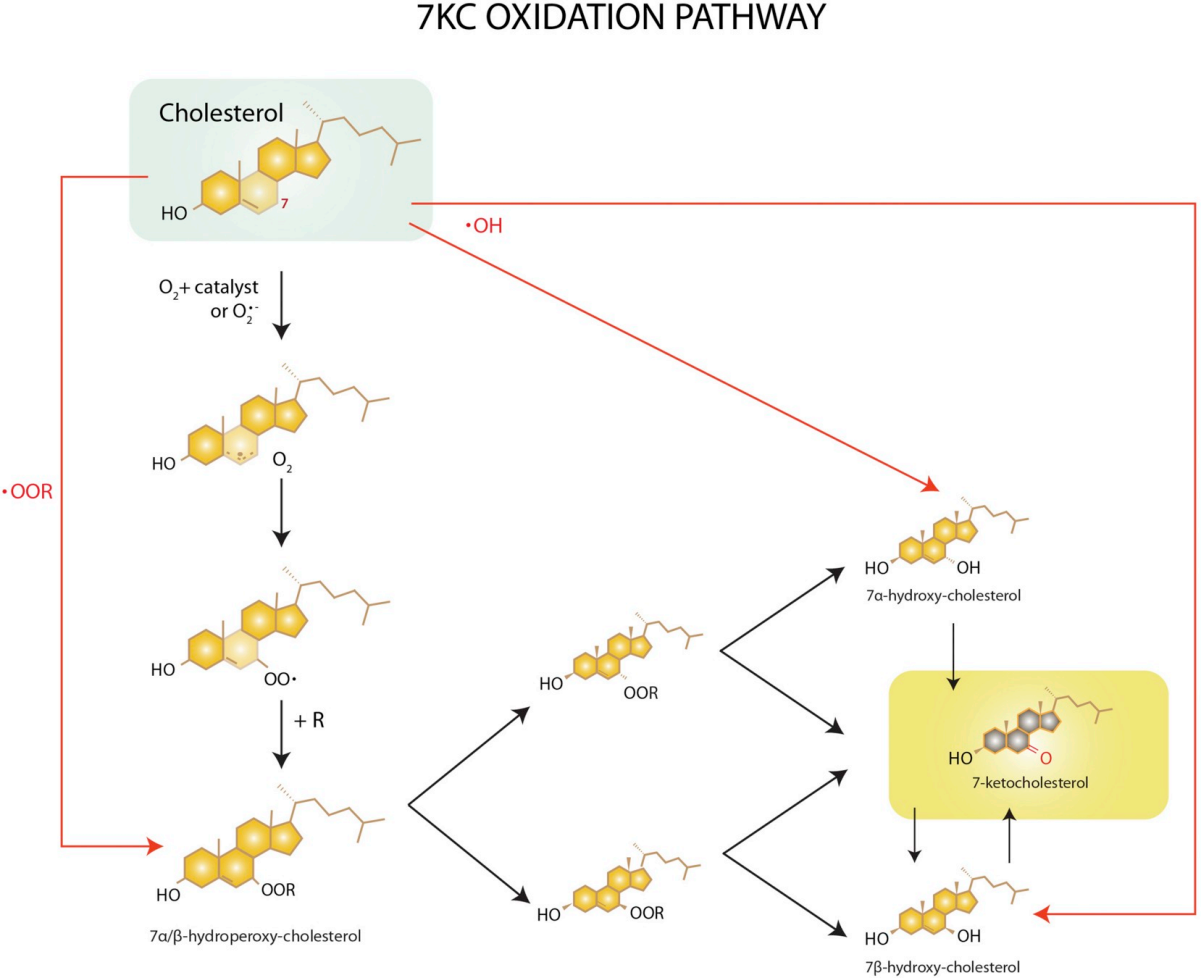
7KC can be nonenzymatically produced via oxidation of cholesterol along multiple different pathways.

Some oxidized cholesterols (oxysterols) are physiological compounds produced enzymatically and serve as signaling molecules, while others are adventitious products of the nonenzymatic reaction of cholesterol with ROS and are generally cytotoxic. Oxysterols are shown to be consistently 10 to 100 times more reactive than native cholesterol, making biological systems quite sensitive to these oxidized sterols [19,20]. Nonenzymatically-produced oxysterols are present in oxLDL, in atherosclerotic plaques, and in all cells to varying degrees; the predominant and most toxic of these is 7KC [21,22] which forms when cholesterol oxidation occurs on the C7 position. Auto-oxidation of cholesterol can occur via the reaction of O2 oxygen, hydroxyl radicals, peroxides, or superoxide catalyzed by metal, radiation, or heat [11,23] (Fig. 1). The 7 position on cholesterol seems to be the most reactive with oxygen and a carbonyl group the most stable form [11]. Hydroxyl and peroxide groups often form first and then further oxidize into 7KC [24]. Consistent with 7KC being the most chemically stable and common auto-oxidized oxysterol in-vitro, 7KC is also the most prevalent nonenzymatically produced oxysterol in-vivo [25].

7KC is also able to convert into 7β-hydroxycholesterol (7β-OHCh) in vivo via 11β-hydroxysteroid dehydrogenase (11β-HSD), and this reduced product is significantly less toxic than 7KC itself [26]. While this enzyme has been shown to interconvert 7KC and 7α/β-OHCh in some animals [26,27], it seems that human 11β-HSD1 in live cells only functions to stereospecifically reduce 7KC to 7β-OHCh in the liver, thus detoxifying and metabolizing it [26]. The production of 7α-OHCh is generally via the 7α-hydroxylase enzyme, which is essential for the formation of bile acids in the liver [28]. 7α and 7β-OHCh are very similar molecules, 7β-OHCh being the more stable of the two, and they both show some cytotoxic effects. However, it appears that the body is able to efficiently process these reduced derivatives [26], possibly because they are more soluble and/or less damaging than 7KC, as 7KC is generally formed by harmful ROS.

A separate mechanism proposed in the formation of 7KC has been suggested to involve the enzymatic conversion of lanosterol to lathosterol and 7-dehydrocholesterol by acetyl-CoA [13,29]. 7-dehydrocholesterol (the immediate precursor of cholesterol) can then be oxidized by cytochrome P450 7A1 to 7KC^13^. This mechanism is more plausible for the formation of 7KC in brain tissues than in other tissues, because of the prominence of lanosterol, lathosterol, and 7-dehydrocholesterol in neurons [30].

As with cholesterol, oxysterols can be present in free-form or in esterified form. Generally, unesterified sterols are found in the plasma membrane while esterified sterols are found within lipoproteins. 7KC does not seem to be efficiently esterified and exported from cells to the lipoprotein cholesterol transport system [31] and is normally present at very low levels in the serum. Exceptions to this in certain diseases are discussed in sections 4 and 6.

7KC can also be found in LDL, but generally only when the LDL itself has been oxidized (oxLDL). 7KC accounts for up to 30% of the total sterol in these oxLDLs [9,32], but it is important to note that free 7KC exerts toxicity differently from oxLDL. Free 7KC seems to predominantly permeabilize the plasma membrane by disrupting calcium channels, while cholesterols in oxLDL are taken up by endocytosis and are ultimately sequestered and stored in the lysosome [33,34].

7KC induces apoptosis in its free form by increasing both extracellular Ca^2+^ influx and intracellular Ca^2+^ mobilization; free 7KC concentrations of 10–30 μM will trigger apoptosis in a variety of cell types [8,17,[35], [36], [37], [38]]. 7KC-induced Ca^2+^ increase also activates cytosolic phospholipase A2 (cPLA2) and releases arachidonate from membrane phospholipids. Using this released arachidonate, 7KC can then be esterified by the enzyme Acyl-CoA:cholesterol acyltransferase (ACAT) to form 7KC-arachidonate [8]. It has been shown that this esterification of 7KC can decrease the esterification of cholesterol [39], which is required for its loading inside LDL. Thus, 7KC can impair normal cholesterol metabolism and exacerbate conditions which already compromise cholesterol metabolism.

Additionally, 7KC increases the production of ROS by activation of NADPH oxidase and triggers an apoptotic stress response [40]. Studies have shown that 7KC has a primary role in intracellular ROS overproduction via stimulation of NADPH-oxidase (NOX). Nearly full protection by multiple selective NADPH-oxidase inhibitors suggests that 7KC can interact directly and quickly with this enzyme to trigger ROS overproduction [18]. 7KC has also been shown to trigger ROS, and ROS alters lysosomal activity which results in diminished mitochondrial turnover [5,16].

7KC can be metabolized in the liver by enzymes like 11β-hydroxysteroid dehydrogenase type 1 (11β-HSD1), which is why it is not acutely toxic to ingest foods high in 7KC. Reductive enzymes like 11β-HSD1 convert 7KC to 7β- and 7α-hydroxycholesterol [26,41,42], while sterol sulfotransferases (specifically SULT2B1b) have been shown to sulfonate oxysterols like 7KC (and its reduced derivatives), making them significantly less cytotoxic [43]. However, these enzymes are largely restricted to the liver, and do not meaningfully metabolize 7KC in other tissues.

In summary, 7KC is produced by the oxidation of cholesterol at the C7 position primarily nonenzymatically by ROS or alternatively by CYT-mediated oxidation. Intracellularly 7KC partitions into membranes in its free form or is esterified for transport in lipoproteins. If 7KC is consumed and absorbed from the digestive tract, 7KC can be reduced by enzymes in the liver or sulfonated elsewhere in the body by sulfotransferases [43,44].

## 3 Biology of 7KC

### 3.1 General cellular effects of 7KC

Cholesterol is one of the most common molecules in the human body – it is found in all cellular membranes and is essential for maintaining elasticity, regulating membrane trafficking, and organizing signaling molecules at the cell surface [42,45]. Likewise, 7KC can be found anywhere that cholesterol can be found, and this has many biological implications. Cholesterol-rich lipid rafts are particularly affected because 7KC creates surface packing defects in the cell membrane, leading to increased permeability due to increased hydrophilicity of the C7 carbonyl group. This increase in permeability causes the opening of calcium channels, allowing calcium to enter the cell and induce apoptosis, and can also inhibit cholesterol efflux [32,34,42,46]. Conversely, the closely related 7β-OHCh does not easily enter and disrupt cell membranes, which likely accounts for its relatively reduced cytotoxicity compared to 7KC [47].

Perhaps the most notable effect of 7KC on cells is its ability to activate NADPH oxidase (NOX), NADPH oxidase (NOX), leading to rapid ROS generation and eventually cell death by apoptosis [18]. 7KC has been shown to promote ROS production in neutrophils by enhancing the translocation of cytosolic NOX components to the cell membrane, where NOX is active to increase ROS production [48]. The oxidative stress induced by 7KC also affects membrane permeability of the lysosomes and other organelles [49]. 7KC-induced apoptosis via NOX-mediated ROS production and oxidative stress, caspase activation, lysosomal degradation, and phospholipidosis has been demonstrated in human promonocytic U937 cells [20,37]. 7KC has also been shown to induce expression of interleukin-1B (IL-1B), IL-6, IL-8, MCP-1, tumor necrosis factor α (TNF-α), and macrophage inflammatory protein-1B (MIP-1B) in human macrophages/monocytes, showing that 7KC can also affect how cells grow and communicate with one another [50]. 7KC can also cause myelin figure formation and polar lipid accumulation; there is strong evidence that this is dependent on the PI3–K signaling pathway [37].

7KC has also been shown to activate the unfolded protein response (UPR) of the endoplasmic reticulum (ER). This pathway is a key homeostatic regulator in cells. The UPR is initiated when the ER is under stress, the canonical stressor being the presence of excess misfolded proteins. This instigates a number of signals which slow down protein synthesis, increase protein folding machinery, and increase degradation of incorrectly folded proteins. If these alterations do not alleviate the ER stress, these same pathways can initiate cell death. Importantly, 7KC is able to perturb the ER membrane, activating UPR and eventually destroying the cell [20,[51], [52], [53]].

Oxysterol sensors, specifically the Liver X Receptor (LXR) and oxysterol-binding-proteins (OSBPs), regulate many of the functions of oxysterols and their roles in regulation of lipid metabolism, cholesterol homeostasis, development, and cell growth. For instance, LXRs control the expression of the ATP-binding cassette (ABC) transporters, and activation of these transporters has implications in tumor growth, immunosuppression, cholesterol metabolism, and apoptosis/phagocytosis [[54], [55], [56], [57], [58], [59], [60], [61]]. Some research suggests that one role for LXR activation is to provide an adaptive response to oxidative stress, therefore protecting cells from further damage. LXR can be activated by multiple different oxysterols, including potentially 7KC. However, the role of 7KC in this pathway is still tenuous. It has been shown that 7KC is a weak agonist for the LXR when LXR-mediated transcription is activated [62]. This would suggest that a cell already responding to oxidative stress could be more affected by 7KC buildup via the LXR pathway. More recently, there is evidence that LXR activation can protect cells from 7KC toxicity, particularly in the brain [57]. It is also debated whether 7KC can effectively bind OSBPs, which are used by the body to transport unesterified oxysterols. However, expression levels of ABCA1 (a key regulator of intracellular sterol levels and sterol efflux) is controlled via both LXR and OSBP, suggesting that oxysterol receptors could make a good target for the control of cholesterol efflux from macrophages [63,64]. Establishing the importance of 7KC in these pathways requires further work, but is suggested as a fruitful avenue of exploration.

Peroxisome proliferator-activated receptor gamma (PPARγ) also regulates expression of ABCA1 genes, inducing cholesterol removal in a transcriptional cascade mediated by the LXR [65]. OxLDLs and especially 7KC have been shown to be activating ligands for PPARγ, resulting in upregulation of proline oxidase (POX), initiating apoptosis [55,66].

PPARγ and 7KC have been linked to poly-ADP-ribose (PARP) formation, which has implications in many age-related diseases including cancer, atherosclerosis, and Alzheimer's disease [[67], [68], [69], [70]]. Furthermore, PARP has been shown to repress LXR expression, possibly counteracting activation of LXR by oxysterols and acting as a negative feedback mechanism to control ABCA1 gene expression in response to cholesterol metabolism and efflux [71]. More research is clearly necessary to fully elucidate the cross-talk between these many different pathways, and the exact role(s) which 7KC and oxysterols play is yet to be determined. However, there are a number of known effects and these are a key subject of this review.

Because the enzymes necessary to detoxify 7KC are mainly expressed in the liver, 7KC in other tissues can remain toxic, leading to ROS generation and increased 7KC formation. Some studies suggest that at least some of these responses are adaptive, where oxidative stress signals trigger an endogenous stress response and kill malfunctioning cells [55,66,72]. Paradoxically though, a significant accumulation of dead cells can cause more harm than good.

From the above it is clear that increased ROS, cell recruitment, and membrane disruption creates a multi-faceted pathway for 7KC-induced cell death. Interference with biological processes including enzymatic reactions, endothelial functions, oxidation of proteins and other molecules, and induction of apoptosis are just a few of the direct consequences from 7KC accumulation in cells. The result is inflammation in many forms, with the potential for causing significant damage to tissues all over the body. This complex combination of oxidation, apoptosis, and autophagy has been dubbed “oxiapoptophagy” [73] and has been observed in multiple types of cells including human monocytes, embryonic kidney cells, macrophages, endothelial cells, smooth muscle cells, as well as mouse embryonic fibroblasts, neutrophils, and oligodendrocytes [43,[74], [75], [76], [77]].

The downstream biochemical and metabolic impacts of any primary toxin are always numerous and diverse, particularly with one as ubiquitous as 7KC and we do not intend to attempt to summarize them all here. The downstream homeostatic impacts of 7KC and other oxysterols have been reviewed elsewhere [20,30,37,41,42,46,58,[78], [79], [80], [81], [82], [83], [84], [85], [86]], however it is worth highlighting the effect of 7KC-induced lysosomal, peroxisomal, and mitochondrial dysfunction.

### 3.2 7KC in the lysosome

7KC is a potent lysosomal poison. It has been shown to alter lysosome function by directly increasing membrane permeability and inducing the buildup of unesterified cholesterol in the lysosome [15,87,88]. Accumulation of cholesterol causes further impairment of the lysosomal membrane which in turn prevents fusion with endosomes or autophagophores [89]. Lysosomal aggregates are highly implicated in many age-related diseases including macular degeneration, neurodegeneration, and atherosclerosis [90].

Additionally, 7KC causes lysosomal dysfunction by inhibiting proteolytic enzymes such as cathepsin D and B [17]. Even if lysosomes are capable of fusing with other vesicles or organelles, 7KC impairs the normal hydrolytic activity of the lysosome, thus inhibiting the turnover/recycling of biomolecules and cellular organelles. The buildup of oxysterols in the lysosome can also lead to the accumulation of ceroid/lipofuscin which is a well-characterized marker of aging in postmitotic cells [91]. Furthermore, disruption of normal lysosomal activity/structure results in decreased mitochondrial turnover and subsequent increase in ROS production from damaged mitochondria that are not efficiently cleared [16]. Lysosomal degradation such as that described here is considered pivotal in accelerating otherwise “normal” cellular decay, resulting in premature deterioration of many related cellular processes due to the inability to process toxins [90,91].

The cholesterol transporter protein ABCG1 has been proposed to exert a protective effect against 7KC in the lysosome. While ABCG1 overexpression fails to rescue cells from 7KC toxicity [92], ABCG1 null macrophages are particularly susceptible to 7KC induced toxicity [93]. It was further shown that ABCG1 can exert a cytoprotective effect by facilitating export of 7KC to HDL [94].

### 3.3 7KC in the peroxisome

The peroxisome is an essential component of the cell for, among other things, the oxidation of fatty acids. Because of this, the peroxisome is also a potentially potent source of ROS in cells [95]. Closely associated with the mitochondria and endoplasmic reticulum, functioning peroxisomes are essential for cellular homeostasis.

Treatment of microglial BV-2 and 158 N cells with 7KC significantly alters the integrity, size, and function of peroxisomes in a dose-dependent manner [96,97]. 7KC treated cells express both p62 and ABCD3 (expression of both of these markers is considered pexophagic) in the peroxisomal membrane, suggesting 7KC triggers selective autophagy of the peroxisome [97].

Dysfunction of peroxisomes has been linked to various diseases, specifically due to downstream unregulated autophagy in the mitochondria and ER [96,97]. While it is unclear whether 7KC-induced autophagy is protective or responsive, it is known that 7KC can increase accumulation of VLCFA (very long chain fatty acids). These fatty acids likely accumulate because the peroxisomes are unable to sufficiently oxidize them, and these VLCFAs are known to favor oxidative stress and cell death [97].

Better models are necessary to fully elucidate the direct and/or indirect effects of 7KC on the peroxisome, but it is clear that the peroxisome plays an important role in 7KC-induced cellular damage and aging [98].

### 3.4 7KC in the mitochondria

One might expect a direct effect of 7KC on mitochondrial function and therefore free-radical release. In fact, 7KC has been shown to cause mitochondrial dysfunction in cultured cells, namely a loss of mitochondrial membrane potential leading to reduced oxidative phosphorylation and reduced ATP production [97]. We see no evidence in the literature, however, that 7KC accumulates or is produced in the mitochondria. This could be due to the fact that mitochondrial membranes are low in cholesterol [99].

Does that mean that the mitochondria plays no role in generating 7KC? As mitochondria are the primary source of ROS and the reaction of ROS with cholesterol is the primary source of 7KC (Fig. 1) this seems unlikely. One possibility is that ROS leaks out of the mitochondria and reacts with cholesterol elsewhere. Another possibility is that the little cholesterol that is present in the mitochondrial membranes does form 7KC readily, but that mitochondria with significant amounts of 7KC are rapidly subjected to mitophagy. In this case the 7KC would bioaccumulate in the lysosome rather than the mitochondria, even if the mitochondria was the source of much of the cell's 7KC. Certainly mitochondria generate ROS, ROS leads to more 7KC, and 7KC causes greater ROS production. To what extent these are direct or indirect phenomena requires greater experimental attention. Perhaps additional work with more sensitive tools will reveal such a role for an interplay between mitochondria and 7KC.

## 4 Pathology of 7KC

It is clear that oxiapoptophagy can occur in many different cell types all over the body. However, cell death has varied pathologic effects depending on the type and location of the cells affected. Thus, 7KC is highly implicated in many different diseases with many different pathologies.

### 4.1 Cardiovascular and arterial diseases

**Fig. 2:**
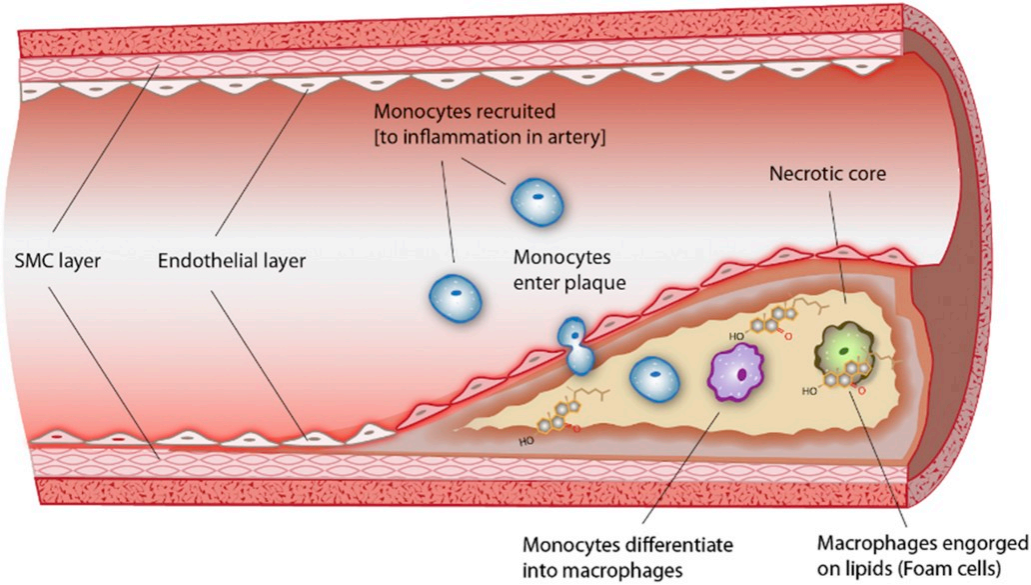
Formation of foam cells and arterial plaque due to 7KC-induced differentiation and buildup of ROS.

Perhaps the most well-known 7KC-related disease is atherosclerosis, the accumulation of cholesterol-laden macrophages (foam cells), lipids, calcium, cholesterol crystals, and connective tissue in the inner layer of the arterial wall [100]. Atherosclerotic foam cells accumulated in plaques also show many characteristics of acquired lysosomal storage disorder [101], which could be attributed to high levels of 7KC (Fig. 2). Additionally, macrophages exposed to oxLDLs accumulate cholesterol esters within their lysosomes. It is thought that cholesterol accumulation causes lysosomes to lose proper pH, so they can no longer efficiently hydrolyze and process cellular debris due to lysosomal proton pump inhibition [102,103]. Anything that blocks or reduces the clearance of sterol from lysosomes can contribute to proton inhibition, thus 7KC accumulation in lysosomes has the potential to exacerbate the disruption of lysosome pH maintenance. As discussed above, 7KC can also activate a stress response via LXR signaling. The LXR is also active in macrophages [96] and thus aberrant LXR signaling due to an excess of 7KC [62,104] could be responsible for part of the mechanism by which 7KC is toxic to macrophages, promoting inflammation in atherosclerotic plaques.

In hypercholesterolemic patients, 57% of the plasma oxysterols are reported to be 7KC, followed by 7-α/βOHC (21%, a direct product of 7KC metabolism). In arterial plaques, 55% of oxysterols are reported to be 7KC, with the second and third most abundant being cholestane-3β,5α,6β-triol and 7-α/βOHC at 13% and 12%, respectively [25]. While it is controversial whether hypercholesterolemia alone is sufficient to elevate circulating 7KC levels [105,106], cross-sectional studies demonstrate that circulating 7KC levels are elevated in patients with atherosclerosis in proportion to the severity of their disease [4,[106], [107], [108], [109]]. Moreover, prospective studies find that higher levels of circulating 7KC are associated with greater future risk of cardiovascular events and total mortality [48,100,110,111]. Highly elevated 7KC in red blood cells (RBCs) is strongly correlated with heart failure [112]. It is notable that the strong disease correlation in heart failure is associated with intracellular 7KC and not serum 7KC levels, as in atherosclerosis.

Endothelial cells form the inner barrier layer of the blood vessel and are key components of normal arterial function. An initiating event in atherogenesis is disruption of endothelial cell function allowing the atherosclerotic plaque to form and accumulate foam cells directly underneath the endothelial layer [5,113]. 7KC is a pro-inflammatory, pro-oxidant, pro-apoptotic, and fibrogenic molecule that alters endothelial cell function by disrupting cell membranes and critical ion transport pathways for vasodilatory response [5,20,27,35,79,114]. 7KC has also been shown to up-regulate adhesive molecules such as ICAM, VCAM, and E-selectin, suggesting that 7KC has an important role in the initial association of leukocytes and monocytes which penetrate through the endothelial barrier, ultimately resulting in atherosclerotic plaque formation [38,115,116].

Due to evidence that 7KC is a significant contributing factor to atherosclerosis, 7KC is also implicated in the most prevalent cardiovascular diseases, including peripheral artery disease, stroke, myocardial infarction, angina pectoris, and coronary heart disease. In these diseases, endothelial cells are crucial because plaques form in the subendothelial space of arteries due to build-up of lipids and foam cells (dysfunctional macrophages); dead endothelial cells themselves also contribute to the plaque [100]. Smooth muscle cells (SMCs), another key component of atherosclerotic plaques, are more susceptible to fibrous cap weakness and destruction when exposed to 7KC. The critical SMC-rich protective fibrous cap of the plaque helps stabilize the diseased tissue. When exposed to 7KC the cap can weaken and rupture. Rupture exposes the underlying tissue to the blood resulting in thrombosis and death of downstream tissue [117]. Apoptosis is also induced in these SMC cells via the caspase-3/7-dependent pathway, ubiquitination, and myelin figure formation, thus adding a third type of cell to the hemostatic blockage [19,101,102]. Laboratory conditions mimicking air pollution (PM 2.5) create a highly oxidative environment which also promotes atherosclerosis that is associated with increased 7KC levels [118].

The uptake of oxLDL by arterial macrophages has also been shown to be pivotal in the formation of these plaques [115,119,120]. It is thought that macrophages are quite capable of processing native cholesterol in LDLs, but the more oxidized LDLs are taken up in an unregulated manner and are not as easily processed, eventually causing macrophages to become dysfunctional and adapt an atherogenic foam cell phenotype. Modified lipoproteins such as oxLDL are taken up by arterial wall macrophages in an unregulated manner via a LDL scavenger receptors as they attempt to clear a plaque [7]. This includes the uptake of oxidized lipids such as 7KC.

Inside macrophages, free 7KC can alter organelles and has been shown to induce a stress response which can result in release of Cytochrome C from mitochondria in the production of ROS and mitochondrial apoptosis [76].

After initial atherogenesis, 7KC does not become less toxic - continuous stimulation of ROS production and activation of the ATM/Chk2 & ATR/Chk1, p53, and PI3K/Akt signaling pathways in vascular endothelial cells alters crucial cell cycle regulation. This also makes 7KC a risk factor for cardiovascular disease in the general population [5]. Samples of patient atheroma have been shown to have increased KDEL and CHOP (transcription factor induced by ER stress/UPR) activity within macrophages and SMCs, indicating high levels of ER stress. These factors were particularly upregulated in samples where the plaque was unstable, indicating that plaque rupture, thrombosis, and vessel occlusion typical of acute coronary syndrome (ACS) may be strongly affected by ER stress and the UPR response. These effects were also seen in 7KC treated CASMCs (coronary artery smooth muscle cells) and THP-1 cells in a dose-dependent and time-dependent manner, suggesting that 7KC-induced stress may be pivotal in not only formation but also rupture of atherosclerotic plaques [53].

7KC has also been shown to affect macrophage polarization, causing more inflammatory M1 macrophages rather than anti-inflammatory M2 macrophages; this increase in inflammation further stimulates expansion of plaques [20,119]. *In vitro*, however, M2 macrophages are somewhat more prone to becoming foam cells than M1 macrophages [[121], [122], [123], [124]]. 7KC is able to recruit more (doomed) monocytes by activating an endothelial cell stress response; monocytes read this as a signal to attack the area affecting the expression of cell adhesion molecules (CAM), which also results in increased inflammation and arterial blockage [50].

#### 4.1.1 7KC in atherosclerotic plaque calcification

7KC and 7KC-induced ROS have also been highly implicated in inorganic phosphate (Pi)- induced calcification of VSMCs by causing lysosomal dysfunction and apoptosis [17,[125], [126], [127]]. In the presence of Pi, higher concentrations of 7KC cause marked apoptosis and extracellular calcification *in vitro* [126,128,129]. It is believed that apoptotic bodies, deposited before calcification occurs, contain large amounts of calcium and act as nucleation sites for blood vessel calcification [130]. In addition, while lower concentrations of 7KC may still induce apoptosis, this concentration range also promotes vascular calcification by disrupting the autophagy-lysosomal pathway (ALP) [17].

ALP is a highly regulated process responsible for the degradation or recycling of proteins and organelles within the cell and has been shown to protect against subsequent Pi-induced calcification [131,132]. ALP is induced in the presence of Pi and inhibition of ALP leads to the release of matrix vesicles which contribute to calcification [131,133]. 7KC quickly induces autophagosome formation in VSMCs [72], but long-term exposure to 7KC appears to alter the function of ALP [17]. Recently, 7KC has been shown to disrupt ALP by impairing lysosome function, thereby inhibiting a protective pathway against calcification [17].

**Fig. 3:**
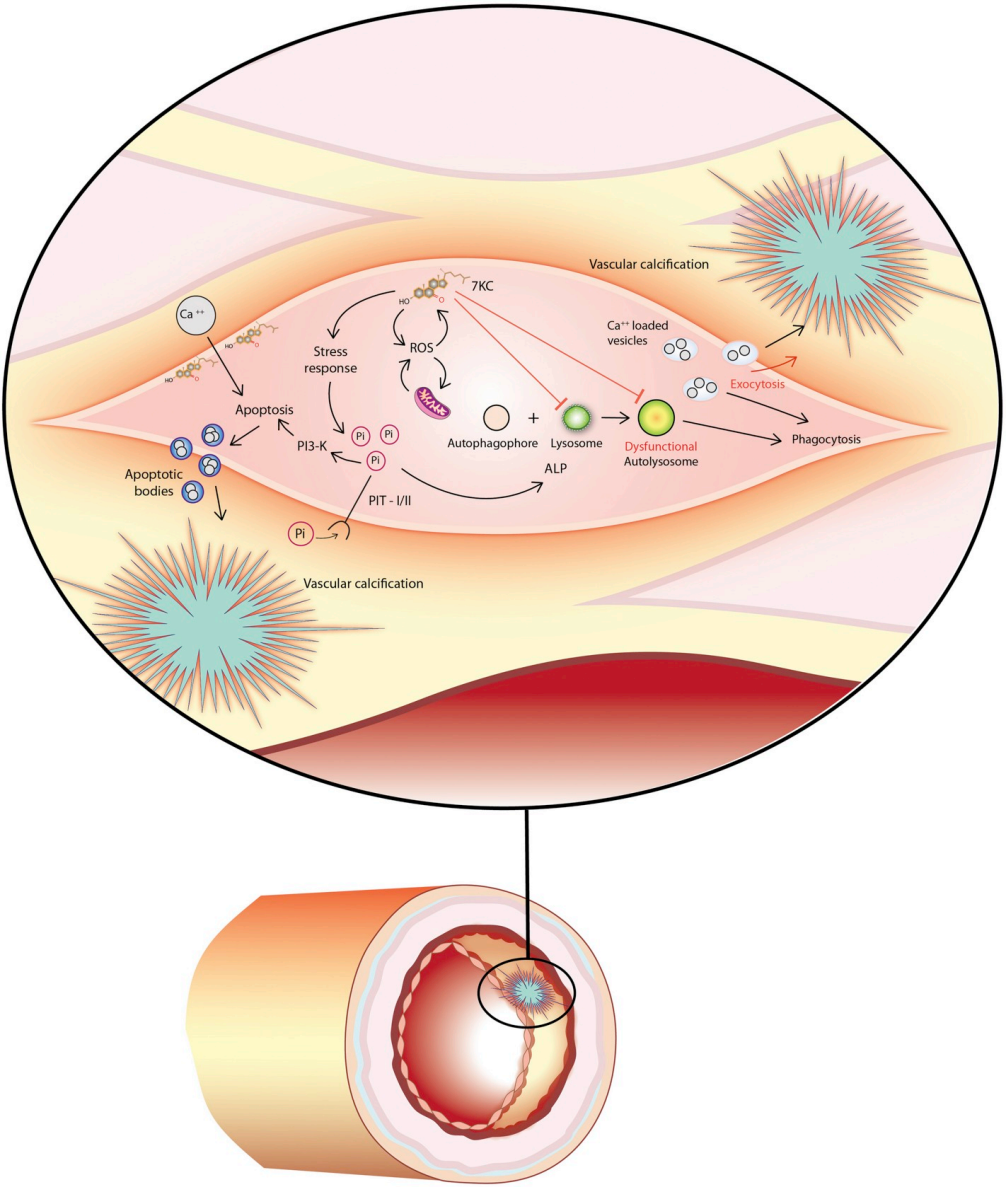
7KC promotes vascular calcification via Pi-mediated apoptosis and disruption of autophagy-lysosomal pathway (ALP) in smooth muscle cells (SMCs).

Recent studies have revealed that calcium deposits related to arterial hardening are triggered by poly(ADP-Ribose) (PAR), which is released when PARP is activated, and that PAR binds to calcium as well as the extracellular matrix in murine MC3T3 osteoblasts and human VSMCs [[68], [69], [70]]. This is strong evidence for the idea that PARP activation, which is already tied to many pathologies, has strong implications in atherosclerosis [70]. Given that 7KC has been shown to increase both calcium and PARP levels [42,[67], [68], [69], [70]], it would not be unreasonable for 7KC to be pivotal in the formation of calcium deposits in arterial plaques (Fig. 3). In summary, the synthesis of many different types of data suggest that 7KC may play a causative upstream role in the progression of atherosclerotic plaque calcification.

### 4.2 Hepatic disorders

The liver is a key organ for maintaining overall homeostasis of the body. Thus, disruptions of liver function can have particularly severe consequences. Although 7KC can be metabolized in the liver by 11β-HSD1, it is still able to interact with receptors, causing oxidative stress, and disrupting membrane rafts and fenestrations in liver sinusoidal endothelial cells [114]. These fenestrations are important for hepatic trafficking, liver regeneration, and interactions between lymphocytes and hepatocytes, so 7KC can seriously affect the livers normal ability to process and filter biomolecules by disrupting membrane rafts.

LXRβ is a ubiquitous receptor but it is also worth noting that expression of LXRα is restricted to the kidney, intestine, fat tissue, macrophages, lung, and spleen. However, LXRα levels are highest in the liver. Both LXRα and β can be activated by 7KC and other oxysterols. Dysregulation of hepatocytic cholesterol homeostasis via LXR and/or OSBP interaction has been shown to be pivotal in the accumulation of hepatic cholesterol and development of non-alcoholic steatohepatitis (NASH) [134].

NAFLD, non-alcoholic fatty liver disease has been characterized as the accumulation of lipids in the liver tissue. Oxidative stress and high levels of fatty acids, cholesterol, and oxysterols, are significant contributing factors to NAFLD, and it has been suggested that the combination of high fat and high cholesterol diets cause hepatocyte ballooning, inflammation, and steatosis due to the accumulation of oxysterols like 7KC [135]. This type of NAFLD (where high levels of cholesterol and fatty acids are present) may be more likely to progress to insulin resistance [136] or steatohepatitis due to cytotoxicity to hepatic cells [135].

NALFD is strongly associated with obesity, type 2 diabetes, and insulin resistance [137], so it is important to recognize risk factors associated with NALFD to prevent further progression of the disease. Hepatocyte apoptosis is known to be a cause of liver damage, causing inflammation and fibrosis if uncontrolled, and thus the accumulation of 7KC in hepatocytes can result in liver damage [135]. Additionally, obesity and insulin resistance are closely linked with adipose tissue inflammation and accumulation of macrophages in fat deposits; all of this can be aggravated by oxysterols such as 7KC [138].

### 4.3 Neurodegenerative diseases

Many neurodegenerative diseases have been linked to oxidative stress, including Amyotrophic lateral sclerosis (ALS) [78,139,140], Parkinson's (PD) [[141], [142], [143], [144]], Alzheimer's (AD) [79,140,145], Huntington's (HD) [146], vascular dementia [79], and multiple sclerosis (MS) [140,147,148]. 7KC has been shown to be highly cytotoxic to neuronal cells and is therefore suspected to be involved in the progression of some, if not all, of these neurological diseases [20,57,85,149]. Oxysterols, unlike cholesterol, can cross the blood brain barrier (BBB) and accumulate in brain tissue [150,151], ultimately causing neurodegeneration. To our knowledge, 7KC's ability to cross the BBB has not been investigated. However, a similar oxysterol, 27-OH, can cross the BBB with similar magnitude to the brain-specific and nontoxic oxysterol metabolite, 24S-hydroxycholesterol [151,152]. 7KC has been found in brain tissue, but it is unclear whether it enters the brain from the periphery and crosses the BBB or if it is created from oxidation of endogenous cholesterol in the brain [153]. Regardless of the source, the presence of 7KC within brain tissue has the potential to cause significant damage.

An alternative or additional hypothesis takes 7KC-induced damage to the BBB into account. Increased populations of oxysterols at the BBB due to an alternate stressor or disease is known to cause damage which affects the integrity of junctions in the BBB [154]. This increase in permeability due to oxidative damage could allow a host of toxic substances to enter the normally protected space of the central nervous system. This would induce another vicious cycle of increased damage, resulting in increased 7KC influx, and ultimately furthering the neurological damage.

7KC-induced oxiapoptophagy has been observed on murine oligodendrocytes and human microglial, neuroblastoma, Jurkat, and bone marrow stem cells [15,57,155,156], so if 7KC can indeed cross the BBB, it could cause significant neurodegeneration. Neurotoxic 7KC molecules have also been shown to inhibit the cholesterol epoxide hydrolase (ChEH) enzyme, which then no longer produces neuroprotective cholestane-3β,5α,6β-triol, increasing 7KC neurotoxicity [85,157].

Research has consistently suggested that oxysterols could be a cause of altered brain cholesterol metabolism, which is an integral part of AD [152,158,159], ALS [139], PD [141,144], and dementia progression [145,160], while multiple studies of oxysterols in post-mortem human AD brains have identified various oxysterols derived from cholesterol autoxidation, including 7KC [5,159,161]. These studies show that there are significant differences in the oxysterol composition of AD [158,159], ALS [139], PD [141,144], and other diseased brain/serum/CSF samples and healthy samples, raising the possibility of an early diagnostic involving sterol concentrations. Chronic epilepsy also shares many of these pathologies, and it has even been suggested that epilepsy is caused by atherosclerosis, so treatment of atherosclerosis could also lessen the effects of epilepsy [162].

Development of AD is perhaps the most well studied neurodegenerative disorder in its relation to 7KC. Marked by the accumulation of extracellular β-amyloid (Aβ) plaques and intracellular neurofibrillary tangles in the brain, AD tangles often include oxidatively modified and hyper-phosphorylated tau (a microtubule assembly protein), which may be increased by molecules that cause oxidative stress, such as 7KC. Aβ plaques are formed largely by the cleavage of the amyloid-β precursor protein (AβPP) into toxic Aβ peptides rather than into non-toxic AβPP peptides. These non-toxic AβPP peptides have been shown to actually protect neurons from radical induced damage [163], while Aβ peptides are instigators of ROS-mediated damage via NOX and mitochondrial dysfunction [79,164], which are provoked by 7KC. AD can be characterized by a vicious cycle of initial oxidative damage to neurons (possibly by 7KC-induced oxidative stress) which leads to promotion of abnormal AβPP cleavage, further instigating more oxidative damage to the cell. In addition to AD, atherosclerosis is an accepted risk factor for vascular type dementia [165]. 7KC has also been implicated in the malfunction of microglia in AD [155,166]. The hypothesis is that 7KC accumulates in the lysosomes of the phagocytic microglia. The lysosomes eventually lose activity and the microglia lose their phagocytic properties. Once the microglia are no longer able to clear extracellular Aβ aggregates the Aβ plaques are able to grow.

Accumulation of 7KC has been found in cerebrospinal fluid (CSF) and plasma of MS patients, and this accumulation appears to be correlated with disease progression and myelin damage [135,136,147]. Accumulation of 7KC has been linked to cerebral atrophy and pathological dysfunction of the BBB [143,154,167].

Diseases such as MS are characterized by neuronal death and axonal damage which is likely tied to demyelination events [67,148]. 7KC is capable of activating microglial cells without the presence of a primary inflammatory stimulus [67] - this induces a similar stress response to what is seen in the arteries. This stress response causes microglial cells to proliferate, migrate, and adhere as they are activated in brain tissue, causing increased neurodegeneration and oxiapoptophagy [67,155,168]. 7KC is capable of entering into the nucleus of microglial cells where it activates poly(ADP-ribose)-polymerase (PARP)-1. This leads to classical M1 activation, triggering the production of inflammatory factors, induction of iNOS, and reduced expression of neurotrophins (nerve growth factors) [67]. At higher concentrations, 7KC can accumulate in microglial cells and become cytotoxic.

Sensorineural hearing loss (SNHL) is closely associated with other neurodegenerative disorders, and is one neurological phenotype of genetic disorders NPC and SLOS (congenital disorders linked to cholesterol homeostasis are discussed in section 4.7) [169] as well as atherosclerosis [170]. It has been suggested that, therefore, cholesterol homeostasis and metabolism are important factors in the progression of this disease as well. This group proposes that hypercholesterolemia triggers stenosis (narrowing) of the spiral modiolar artery, leading to cochlear ischemia and finally SNHL, although it has not yet been studied in depth [82].

Amyotrophic lateral sclerosis (ALS) is also aggravated by oxidative and mitochondrial stress and it has been shown that cholesterol metabolism is defective in ALS patients [139,171]. Oxysterols are therefore highly implicated in ALS, with multiple studies indicating that oxidative stress is significantly increased in ALS postmortem tissue compared with controls, however it remains unclear what the ultimate source of this oxidative stress is [78,139].

### 4.4 Ocular disorders

Given the cytotoxicity of 7KC to other cells, it is not surprising that retinal pigment epithelium (RPE) cells are also subject to 7KC-induced cell death via the release of cytokines, activation of caspase-8 and caspase-12, and mitochondrial damage by induction of oxidative stress [172,173]. Because 7KC clearly causes degeneration of retinal cells, it is logical that 7KC may play a role in causing diseases of the eye including age-related macular degeneration (AMD), choroidal neovascularization (CNV), glaucoma, and cataracts [161,168,[172], [173], [174], [175], [176], [177], [178]]. 7KC is also seen in high levels in retina with photodamage [179].

Macular degeneration occurs when the macula, the central region of the retina responsible for keen vision, deteriorates. Often this deterioration is associated with age, as it can occur slowly over many years before a patient begins to notice central vision loss. Drusen, fatty yellow deposits beneath the retina, accumulates with age and its formation is usually monitored by ophthalmologists; high levels of drusen is known to be an early sign of AMD, particularly when associated with the Bruch's membrane (BM: the innermost layer of the choroid between the retinal pigment epithelium (RPE) and the fenestrated choroidal capillaries of the eye) [180]. The BM acts as a molecular sieve and tends to calcify, fragment, and accumulate drusen with age, particularly in cases of AMD [181].

Studies of 7KC localization in the retina of primates show that unesterified 7KC is mainly located in drusen in the choriocapillaris and BM, indicating that 7KC may be highly implicated in the accumulation of drusen, deterioration of the BM, and therefore in the development of AMD [176]. Retinal microglial cells readily internalize 7KC from culture, changing the retinal microglial phenotype to one which promotes neurotoxicity, angiogenesis, and neuroinflammation [168]. In addition, oxysterols including 7KC have been shown to induce inflammation in primary porcine RPE cells via induction of ROS overproduction [182].

In rat eyes, direct intravitreal injection of 7KC induces panretinal degeneration within one week [183]. More recently, this method has been used to show that 7KC treatment decreases the number of apical microvilli of RPE cells and, moreover, detaches the microvilli from the outer segment, suggesting hampered phagocytosis of photoreceptor outer segments (POS), an essential function for maintaining eye health. In ARPE-19 cells, this has been shown to occur via the ERK signaling pathway [184].

It has been suggested that initial 7KC accumulation in the eye may also induce microglial translocation to the outer retina, so these neurotoxic, angiogenic, inflammatory microglia can activate changes which in turn advance AMD [175]. It has even been shown that oxidative damage to the choriocapillaris of mice leads to very similar pathogenesis of AMD, suggesting at the very least that oxidative stress contributes to the progression of AMD [175,178].

Some evidence suggests that oxysterols and 7KC are implicated in the development of other retinal pathologies, but the exact role of 7KC is yet to be determined. Studies on human RPE and VEC cells show that 7KC can induce VEGF (vascular endothelial growth factor) via the LXR, which is also triggered by hypoxia and is known to be involved in choroidal neovascularization (CNV) [46,176,177]. 7KC itself has been found to be present in quantifiable amounts in cataractous lenses (4.2±0.32 mM/mol cholesterol) [174]. It is known that cholesterol metabolism, lipid raft formation and signaling, and oxysterol formation are all important processes in the formation of retinopathies, but it is unclear how or if specific oxysterols can alter the cellular environment and encourage ocular disease progression [46].

### 4.5 Pulmonary diseases

Idiopathic pulmonary fibrosis (IPF) is an aggressive lung disease with no effective therapies; IPF is marked by accumulation of myofibroblasts in lung tissue, causing fibrosis. Expression of NOX4 and ROS production significantly increases fibrosis in multiple organs, but pulmonary fibroblasts show particularly high expression which correlates with ROS levels [[185], [186], [187], [188]]. This indicates that ROS generation via NOX4 (a known effect of high 7KC levels) drives fibrin formation and accumulation, leading to epithelial cell death and fibrosis [185,186,188].

7KC and 7KC-related ROS accumulation may also have implications in COPD, where oxidative stress overrides the body's natural antioxidant defenses and results in chronic inflammation and infection [189]. The disarrangement of fatty acids and oxidative stress are also key factors of cystic fibrosis, and it has been found that 7KC is particularly elevated in diseased patients with cystic fibrosis over control subjects [190].

Full elucidation of 7KC's role in pulmonary diseases is yet to be accomplished, but it does appear that 7KC has pathological effects on lung tissue and cells.

### 4.6 Gastrointestinal diseases

The intestinal mucosa are essential for proper digestion - if they are too permeable or defective, the mucosa has been shown to be implicated in inflammatory bowel disease (IBD, a precancerous condition), Crohn's disease (CD), and ulcerative colitis (UC). All of these diseases are intensified by immune and inflammatory responses, such as those caused by 7KC. It has been proposed that oxysterols like 7KC contribute to intestinal inflammation by direct stimulation of inflammatory pathways and by indirect increase of the luminal antigens available to mucosa via disruption of the intestinal barrier [191,192]. 7KC has also been shown to up-regulate the lipopolysaccharide-binding protein CD14 in the intestines, which mediates the bacteria-related inflammatory response in pro-tumoral macrophages [192].

The endothelial wall of the intestine is essential for absorption of nutrients and the maintenance of the body's digestive system. Incubation of human colonic epithelial cells with 7KC has shown enhanced production of ROS via NOX hyperactivation, leading to apoptotic and eventually necrotic cell death in a concentration-dependent manner [191,193]. 7KC has also been shown to decrease epithelial barrier functions by modifying anti-inflammatory IL-10 expression in a co-culture of human intestinal epithelial and dendritic cells [192]. Oxidative stress of gastrointestinal epithelial cells has implications in many gastrointestinal diseases as excessive ROS damage can increase gut permeability, a key cause of many gastrointestinal pathologies.

Induction of ulceration is known to be caused by a combination of ROS-mediated damage and apoptosis which results in cell proliferation arrest. Both IBD and Crohn's are characterized by inflammation of the mucosal layer of the gut, an area where cells are known to respond to 7KC with inflammation [192]. In some cases, persistent ROS causes proto-oncogene activation and tumor suppressor gene mutations in gastric tissues, implicating 7KC in some gastric cancers, including colorectal cancer (CRC). This happens when free radicals, a result of ROS, convert dietary procarcinogens into carcinogens [194].

Inflammation of the gut and gastrointestinal tract is known to be exacerbated by, if not caused by, oxidative stress. Oxidative stress results in gut permeability, leading to the progression of many gastrointestinal disorders [[191], [192], [193], [194]]. As 7KC is known to increase ROS production, reduction of 7KC should lessen the oxidative stress of gut epithelial cells.

### 4.7 Congenital disorders

Niemann Pick Disease (NPD) types A, B, and C; Infantile Neuronal Ceroid Lipofuscin (NCL or Batten's Disease); Gaucher's Disease; Lysosomal Acid Lipase (LAL) Deficiency; Familial Hypercholesterolemia (FH); Cerebrotendinous xanthomatosis (CTX); Smith-Lemli-Opitz Syndrome (SLOS); Sickle Cell Disease (SCD); and X-linked adrenoleukodystrophy (X-ALD) are all genetic diseases linked to cholesterol metabolism in some way. These diseases are marked by imbalanced cholesterol homeostasis, with a wide variety of symptoms from early death to neurodegeneration to heart disease - here we will discuss the known and potential involvement of 7KC in these congenital disorders.

Niemann Pick types A (NPA) and B (NPB) result in the deficiency of acid sphingomyelinase (ASM), which functions in the lysosomes to metabolize sphingomyelin, causing its accumulation in cells. NPA is generally fatal by age 5 as it progresses neurologically [195], while NPB patients usually survive longer but with enlarged livers or spleens, often leading to heart disease and respiratory problems [196].

Niemann Pick Type C (NPC) is characterized as an inability to clear cholesterol or other lipids from lysosomes. This causes the accumulation of cholesterol and oxysterols within cells. The liver, spleen, and brain are the key organs affected [106,152,197,198]. One manifestation of this cholesterol accumulation is a reduction of ASM activity, so all three Niemann Pick disorders are considered related. NPC is always fatal, usually by age 20, and causes vertical gaze palsy, enlarged liver, enlarged spleen, and/or jaundice before neurological symptoms appear [[106], [152], [197], [198]]. NPC is also unique in that plasma oxysterols correlate negatively with the patient's age and positively with serum total bilirubin, suggesting the involvement of the liver in the elevation of oxysterols [199].

NPD and Gaucher's diseases are lysosomal storage diseases caused by genetic mutations. NPD is caused by mutations in the NPC1, NPC2, or SMPD1 genes [196,197] while Gaucher's is caused by mutations in the GBA gene, resulting in deficiency of glucocerebrosidase [200]. Both of these diseases cause significant damage to organs and tissue due to the abnormal accumulation of fatty molecules [197,200], and both of these diseases show significant increases in plasma 7KC compared to normal [199,201]. For NPC, a positive correlation between the 7KC profile and the severity of the disease can be seen [105], however the possibility of 7KC as a biomarker for this disease has been refuted due to false-positive results from cholestasis and/or biliary atresia [202].

As NPC is related to a buildup of cholesterol, some work has been done with cholesterol binding molecules called cyclodextrins in an attempt to alleviate the symptoms. It seems that hydroxypropylated cyclodextrins show some specificity for 7KC over cholesterol, and these seem to work effectively to rescue *in vitro* cultured foam cells [100], NPC-1 derived cells [203], mice [203], and cat models [204] of NPC, but the efficacy in humans remains to be seen [205].

7KC has also been found in high levels in patients with infantile neuronal ceroid lipofuscinosis (NCL or Batten disease) [105,199]. This is a rare, progressive neurological disease caused by mutations in the CYP27 gene which is responsible for converting cholesterol to cholate and chenodeoxycholate. The mutation results in high serum cholestanol and severe neurological symptoms like seizures and dementia [206]. It is not unlikely that high levels of cholesterol in high-stress conditions would also oxidize and result in high levels of 7KC, causing more stress to the already-compromised nervous system and progressing the disease.

Lysosomal acid lipase (LAL) deficiency can present itself in varied severity and thus underlies many cholesteryl ester storage disease (CESD) phenotypes, but all LAL mutations lead to accumulation of cholesterol esters and triglycerides in lysosomes. Symptoms of LAL deficiency can range from early death before six months (with total deficiency) to hypercholesterolemia, hyperlipidemia, and/or atherosclerosis [207]. Patients with LAL deficiency have been found to have abnormally high levels of 7KC in plasma, perhaps due to a compensatory upregulation of HMG-CoA reductase to increases endogenous cholesterol biosynthesis (due to low free cholesterol), increasing the formation of oxysterols [199,208,209] Increased formation of oxysterols further increases oxidative stress, worsening the condition.

It should be noted that studies have reported varied results for 7KC levels in patients with familial hypercholesterolemia (FH). Some studies found FH patients to have 7KC levels within the control range, while others show an increase in plasma 7KC concentration [208]. It has been suggested that 7KC could be a useful biomarker of certain congenital disorders, but FH may be an exception to this as FH is due to mutations in the LDL receptor gene, resulting in high blood cholesterol and relatively low intracellular cholesterol [199]. However, some studies do suggest that oxysterols are significantly increased in FH, which is logical as an increase in cholesterol concentration would imply an increase in oxysterols as well [208].

Cerebrotendinous xanthomatosi (CTX) results from the abnormal storage of lipids due to a lack of cholesterol-metabolizing enzyme sterol 27-hydroxylase. This causes neurological problems due to increasing xanthomas (fatty deposits) in the brain, resulting in peripheral neuropathy, dementia, dysarthria, and more [210]. CTX patients have been shown in multiple studies to have increased 7KC levels [80,105], and it has been suggested that this may be due to the upregulation of cholesterol 7α-hydroxylase. This could ultimately result in increased 7KC levels by conversion of 7-dehydrocholesterol to 7KC, but this only happens when cholesterol 7α-hydroxylase is upregulated due to deficiency of sterol 27-hydroxylase [211].

Smith-Lemli-Opitz syndrome (SLOS) has effects all over the body due to a mutation in the DHCR7 gene, resulting in dysfunctional 7-dehydrocholesterol reductase. This leads to an inability to produce cholesterol and therefore the build-up of other, toxic lanosterol byproducts such as 7-dehydrocholesterol, which can convert to 7KC, as previously discussed in section 1. Indeed, 7KC accumulation has been linked to SLOS [80,105,199], although it is still unclear how SLOS symptoms, such as nose shape and ptosis (droopy eyelid), are linked to deficient cholesterol metabolism. It has been suggested that cholesterol metabolism may play an important role in vertebrate embryogenesis, which could result in these strange symptoms [212].

Sickle cell disease (SCD), a red blood cell disorder that causes deformed red blood cells, is associated with curiously low levels of serum cholesterol [213,214]. It has been suggested that this could be due to a reduced blood volume, but it is more likely that this is due to strong down-regulation of cholesterol biosynthesis in SCD patients [214]. SCD has also been shown to increase oxidation susceptibility of LDLs [215], while oxysterols (particularly 7KC) have been found to be increased in sickle RBC membranes compared to normal [216,217]. It has also been shown that these oxysterols can cause direct deformation of sickle RBCs [218]. This suggests that even if oxysterols are not the cause of SCD, some of the symptoms may be relieved or progression of the disease may be slowed by removal of 7KC from these damaged membranes.

7KC is also found in high levels in the plasma of people affected by X-ALD, a genetic disorder where the ABCD1 gene is mutated, so that long-chain fatty acids accumulate in tissues and plasma. High levels of 7KC could induce peroxisomal dysfunction in microglial cells, intensifying brain damage for X-ALD patients [96].

### 4.8 7KC in aging

Oxidative stress has long been causatively implicated in the aging process [219,220]. As described in section 2, 7KC is the most common stable product of a reaction between cholesterol and a free radical. This is a vicious cycle as 7KC also leads to increased free radical production and release, seemingly by plasma membrane permeabilization. As discussed above, mitochondrial dysfunction and free-radical formation are also strongly implicated in the aging process and so while the precise mechanistic links between mitochondria, 7KC, and aging are still being elucidated they seem likely to be intertwined.

Is 7KC a biomarker of aging? As discussed in section 4, 7KC accumulation is directly implicated in many diseases of aging, including atherosclerosis, heart failure, AMD, NAFLD, and AD. It is thus reasonable to hypothesize that when otherwise unrelated diseases of aging share a common cause, that this cause is likely to be a part of the biological aging process. 7KC is known to accumulate in phagocytic cells such as macrophages (promoting the formation of foam cells), RPE cells, and microglia. It has also been suggested that *c. elegans* subjected to 7KC could be a good model of 7KC-dependent aging [221]. As 7KC is broadly toxic, and most cells seem to have difficulty metabolizing it, it may be that, with age, 7KC is bioaccumulating and impairing functional activity of the cells and tissue.

As described in Section 3, 7KC is a potent inhibitor of lysosomal function. Lysosomal dysfunction is part of the degenerative aging process and is implicated in the cause of diseases such as AMD, atherosclerosis, and AD in which accumulation of non-degradable biomolecules in the lysosome prevent phagocytic cells from efficiently metabolizing phagocytized biological material [91]. We therefore propose that 7KC plays an important role in the loss of function associated with aging and the lysosomal dysfunction produced by 7KC is a key mediator of this role. Thus, as argued in the previous sections, although diseases of aging present with disparate symptoms, a central element to many of the symptoms could be production and accumulation of 7KC. Despite the fact that the biological effects of 7KC have been studied since at least the 1940s, relatively little has been published quantifying intracellular 7KC levels in vivo with age. This is likely due to the challenges currently associated with accurately measuring 7KC in cells and tissues.

Could intracellular 7KC levels be a biomarker of aging in certain cell types? RBC 7KC levels tentatively seem to be better correlated with heart failure than with age [112], but an independent aging cohort of any cell type has not been performed to our knowledge. More work should be done to investigate the contribution of 7KC to the diseases and disabilities of aging.

### 4.9 7KC in diagnostics and medicine

7KC has been proposed as a therapeutic target for various diseases including atherosclerosis, Alzheimer's, and Niemann Pick type C [100,152]. 7KC is significantly increased in these and other diseases compared to normal, and it is believed that 7KC accumulates in cells of various types and causes damage which results in various pathologies related to these diseases. This buildup of 7KC impedes normal cholesterol metabolism and, perhaps more importantly, significantly worsens conditions which already demonstrate compromised cholesterol metabolism pathway(s). Studies have also linked high 7KC levels with risk for cardiovascular events and/or mortality [48,110,111], and particularly high intracellular 7KC levels in RBCs has been strongly correlated with heart failure [112]. Additionally, 7KC is found in high concentrations in deposits in the eyes of AMD patients in the form of drusen, in the arteries of atherosclerosis patients in the form of plaques, and brains of patients with various neurodegenerative diseases [110,178,199]. This significant alteration of 7KC levels in diseased states compared to normal implies that testing 7KC levels could be a good indicator of disease.

This significant alteration of 7KC levels in diseased states compared to normal implies that testing 7KC levels could be a good indicator of disease. Serum 7KC testing is currently available as a diagnostic, but it is not generally well correlated with disease state or severity [199]. On the other hand, blood cell and tissue 7KC load has been correlated with several congenital diseases of cholesterol metabolism, as discussed in section 4 above. This suggests that a diagnostic measuring intracellular rather than extracellular 7KC might prove to be a valuable clinical tool for multiple pathologies. Notably, such a diagnostic would require some moderately sophisticated processing, and clinical measurement of samples by mass spectrometry remains costly. For this reason, such a diagnostic might not be financially feasible in the clinic at this time without a strong medical justification such as 7KC targeted therapies. Recently 7KC-specific antibodies have become available [168], so perhaps antibody-based testing of clinical samples will one day be possible.

7KC has been tied to numerous pathologies and has no known beneficial biological purpose in higher eukaryotes. Thus, if technologies could be developed to specifically remove 7KC from biological systems there should be no detrimental effects on such treated organisms. Such treatments could potentially alleviate both the direct effects of 7KC toxicity (such as lysosomal dysfunction and membrane permeabilization) as well as the indirect, downstream responses linked to inflammatory stress responses caused by 7KC toxicity. Even in extremely oxidative conditions, 7KC is present in relatively low levels compared to cholesterol [36,39], and cholesterol is a molecule that is relatively easily replenished through biosynthesis or diet and thus off-target effects on cholesterol (and perhaps other beneficial oxysterols) might be reasonably well tolerated, even if the agent is moderately specific for 7KC.

While dietary 7KC is not currently considered toxic and is generally not a regulated food ingredient, it has not been tested in liver disease to our knowledge. As the accumulation of 7KC in hepatocytes can trigger oxidative stress, inflammation, and cell death [222], it seems logical that diseased hepatocytes would be more susceptible to 7KC-induced cell damage such as that discussed throughout this section. Thus, it is suggested that even high dietary 7KC intake could act to exacerbate an already existing liver disease.

### 4.10 Conclusion/summary

In conclusion, it has yet to be demonstrated that 7KC has any beneficial role in the body of animals, and it is clear that 7KC causes cellular damage and causes multiple stress-related responses. 7KC is normally formed through a nonenzymatic reaction between cholesterol and ROS. Once formed, 7KC is fairly stable and difficult to clear. Within cells, it has the ability to significantly stimulate ROS production and eventually apoptosis due to cellular dysfunction. The ability of 7KC to induce a unique process of cell death known as oxiapoptophagy makes 7KC highly toxic to cells both directly by interfering with membrane integrity and indirectly by increasing ROS production.

When fully integrated into membranes, 7KC can directly create permeability due to the presence of a destabilizing carbonyl group, forcing lipid groups on the membrane farther apart. 7KC also directly and indirectly damages organelles including the mitochondria, ER, peroxisomes, and lysosomes; and it is implicated in various diseases associated with aging.

ROS naturally occurs due to normal cellular processes and reacts with native cholesterol to form 7KC. The body cannot process 7KC, and as we age, it bioaccumulates in the lysosomes, particularly of phagocytic cells, accelerating the production of ROS (in conjunction with additional stressors from the environment and possibly related disease states). This vicious cycle accelerates the process of aging and leads to various age-related diseases.

7KC is the most abundant oxysterol in both oxLDL particles and atherosclerotic plaques, indicating the significant role 7KC plays in the progression of atherosclerosis. 7KC has been shown to induce macrophage reprogramming, foam cell formation, and oxiapoptophagy in a multitude of cell types. In atherosclerotic plaques, this results in the deposition of calcium-laden apoptotic bodies, leading to subsequent calcification of the blood vessel.

It has been shown that oxysterols are likely a cause of altered brain cholesterol metabolism which is an integral part of AD, Parkinson's, and other aspects of neurological aging. It is not yet fully understood whether 7KC can cross the BBB, but 7KC is highly toxic to neuronal cells and should certainly form spontaneously inside of them with age. Additionally, 7KC is implicated in macular degeneration as it is a major component of the drusen within the retina. 7KC can also damage the liver by disrupting membrane rafts and fenestrations. Lastly, 7KC is also characterized in congenital disorders such as sickle cell, Niemann Pick, and other lysosomal storage disorders. Ambiguous links between many of these diseases, particularly atherosclerosis and neurodegeneration, further implicates 7KC as an unexplored target in many diseases.

Studying the effects of 7KC on various diseases and pathological states can prove difficult due to differences in how various species or cell lines respond to 7KC. Additionally, certain diseases are nearly impossible to model *in vitro* due to the nature of the disease and complex interactions throughout the body. Thus, it has been suggested that alternatives to animal or *in vitro* models may be useful for studying these effects, such as organoids and microfluidic associated technologies (organ-on-a-chip or body-on-a-chip models) [223]. These technologies could prove invaluable for mimicking living systems accurately based on stem cells and precise engineering. Systems like these have enormous potential for screening drugs, creating personalized models, and elucidating pathophysiology in ways that are simply not possible with living animals or cell culture, particularly in the debilitating diseases associated with 7KC [221]. While these next-generation models may still be in their infancy, it is crucial to begin proving concepts, building databases, and raising awareness and trust in these innovative systems.

We propose that 7KC could be an effective therapeutic target due to its implication in a wide variety of diseases. Although the abundance of 7KC has not yet been strongly correlated to aging or the severity of different pathologies, there is clear evidence to show its destructiveness in biological systems. As more studies are conducted on toxic oxysterols in aging and disease, we hope that more will become known about 7KC abundance in different cells and tissues. This would increase the potential of 7KC as a therapeutic target for various diseases, especially those specifically associated with aging. Considering nonenzymatic oxysterol accumulation, particularly 7KC, as an integral factor in disease progression could change the way we identify and treat these diseases, offering new and possibly broadly effective therapeutics.

## Declaration of competing interest

The authors would like to disclose that Matthew O'Connor and Amelia Anderson have a founding interest in Underdog Pharmaceuticals LLC. This company is developing drugs that target toxic cholesterols such as 7-ketocholesterol.
